# The Role of Antenatal Ultrasound in the Detection of Goldston Syndrome: A Case Report and Review of Literature

**DOI:** 10.7759/cureus.72201

**Published:** 2024-10-23

**Authors:** Isha Gupta, Ashish Gangwal, Rahul Sharma, Mukesh Mittal, Naima Mannan

**Affiliations:** 1 Department of Radiodiagnosis, Sawai Man Singh (SMS) Medical College, Jaipur, IND

**Keywords:** antenatal ultrasound, cerebro-renal syndrome, dandy-walker malformation, enlarged dysplastic kidneys, goldston syndrome, meckel-gruber syndrome

## Abstract

Goldston syndrome (GS) is an extremely rare syndrome involving the central nervous system and kidneys. It is believed to have a familial association and an autosomal recessive inheritance and is characterized by the concomitant occurrence of cystic dysplastic kidneys and Dandy-Walker malformation. We report a case of antenatally detected GS at 22 weeks of gestation in a female with a consanguineous marriage. Meckel-Gruber syndrome and Miranda syndrome are the other inherited cerebro-renal syndromes, which are more common than GS, and differentiating them requires thorough knowledge about the imaging features and associations of these diseases. GS has a poor prognosis, which makes early antenatal detection crucial for appropriate counseling of the parents and for deciding further management strategies. Through this case report, we aim to highlight the crucial identifying ultrasound features of this rare disease and describe a stepwise approach to differentiate GS from other, more common differentials.

## Introduction

Familial inherited cerebro-renal syndromes are a rare group of disorders affecting the kidneys and the central nervous system [1]. Meckel-Gruber syndrome (MGS) is the most common and well-known syndrome in this group while Goldston syndrome (GS) is an extremely rare disorder [2], with our case being the eighth antenatally diagnosed and reported case report all over the world, as per the available literature [3]. It is characterized by cystic renal dysplasia with Dandy-Walker malformation (DWM) [4,5]. Its poor prognosis makes it essential to identify this syndrome antenatally to guide appropriate and timely management. Identifying the sonographic features of this syndrome is important so that radiologists and clinicians do not miss this rare congenital disorder. We present a case of early fetal GS detected at 22 weeks of gestation by antenatal ultrasound, describing the key identifying features, differentials, and further management.

## Case presentation

A 26-year-old Gravida-3 Para-1 female of a low socioeconomic background with a consanguineous marriage presented with a history of amenorrhea for five months for a routine anomaly scan. She had one healthy live female child of four years of age and a history of one spontaneous abortion at eight weeks of gestational age (GA) one year back. There was no family history of renal disease in the parents and no history of congenital abnormalities in previous pregnancies. No history of teratogenic exposure, maternal diabetes, or hypertension was noted. In this pregnancy, she had undergone a prior ultrasound at seven weeks of gestation for confirmation of pregnancy and no ultrasound scan was performed at 10-13 weeks. In the present scan, a single live fetus with femur length and abdominal circumference corresponding to 22 weeks of gestation was identified, with an enlarged head circumference and biparietal diameter corresponding to 28 weeks of gestation (Z score greater than three standard deviations above the mean and lying above the 99 centile) (Table 1) [6].

**Table 1:** Fetal biometry and analysis a: Z-score and centile values are calculated using the fetal growth assessment calculator of the Fetal Medicine Foundation [6]. b: The normal range for fetal kidney length for gestational age is taken from the article by Kiridi et al. [7].

Parameter	Value	Interpretation
Biparietal diameter	66 mm (27 weeks GA)	Increased
Head circumference	260 mm (28 weeks GA)	Increased (Z score- 6.325, >100 centile)^a^
Abdominal circumference	171 mm (22 weeks GA)	Normal (Z score- 0.264, 61 centile)^a^
Femur length	38 mm (22 weeks GA)	Normal (Z score- 0.498, 70 centile)^a^
Estimated fetal weight	552 g	Increased (Z score- 2.159, >99 centile)^a^
Left kidney	Length: 37.6 mm	Increased (Normal range- 24.0 ± 2.5 mm)b
	Width: 20.9 mm	Increased (Normal range- 14.0 ± 1.1 mm)^b^
Right kidney	Length: 36.9 mm	Increased (Normal range- 26.0 ± 1.4 mm)^b^
	Width: 16.7 mm	Increased (Normal range- 16.0 ± 1.1 mm)^b^

**Figure 1:**
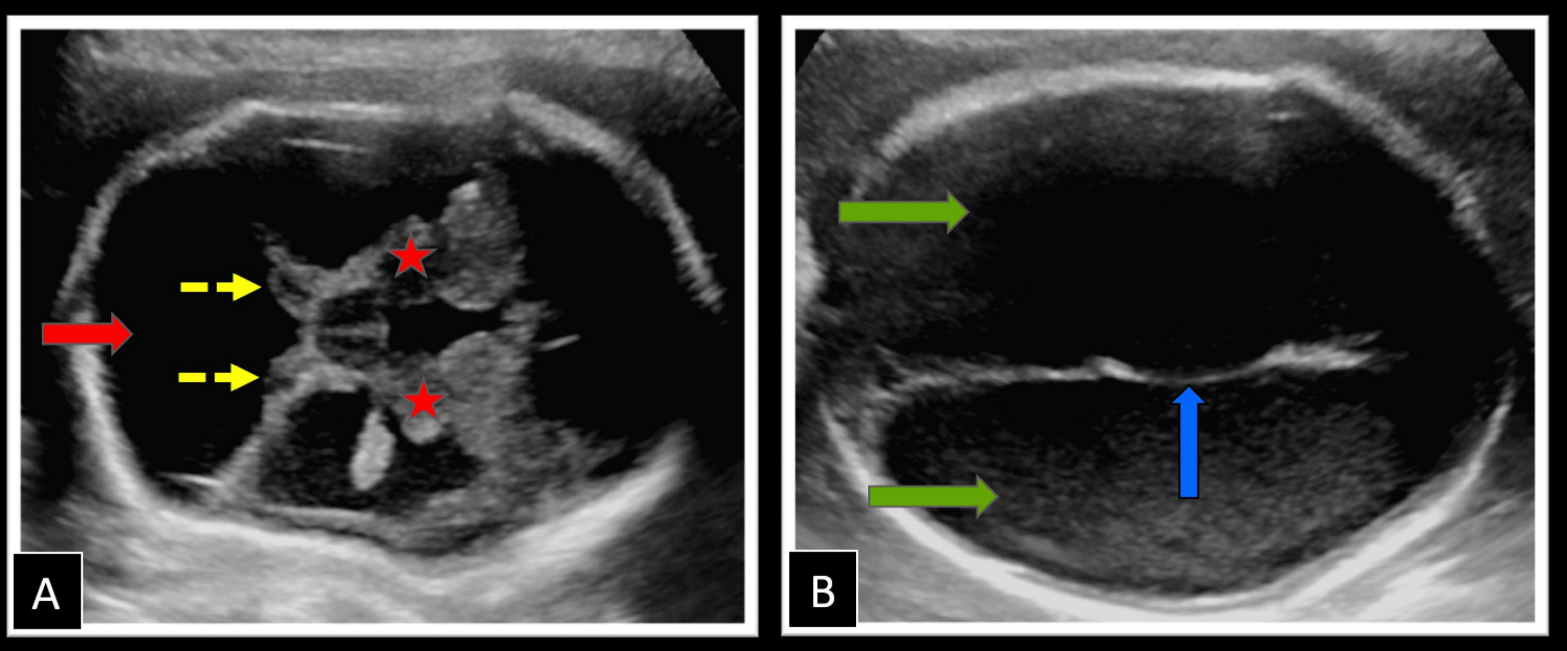
Ultrasound examination of the fetal central nervous system showing the presence of Dandy-Walker malformation (A) Transverse ultrasound image of the fetal brain showing an enlarged posterior fossa with a cerebrospinal fluid containing cyst (red arrow) communicating with the fourth ventricle, with hypoplastic bilateral cerebellar hemispheres (yellow arrows) with vermian aplasia. Also note the normal thalami (stars) with no evidence of fusion and a dilated third ventricle between them. (B) Transverse section through the fetal skull revealing bilateral dilated lateral ventricles (green arrows) with a normal midline falx (blue arrow).

**Figure 2:**
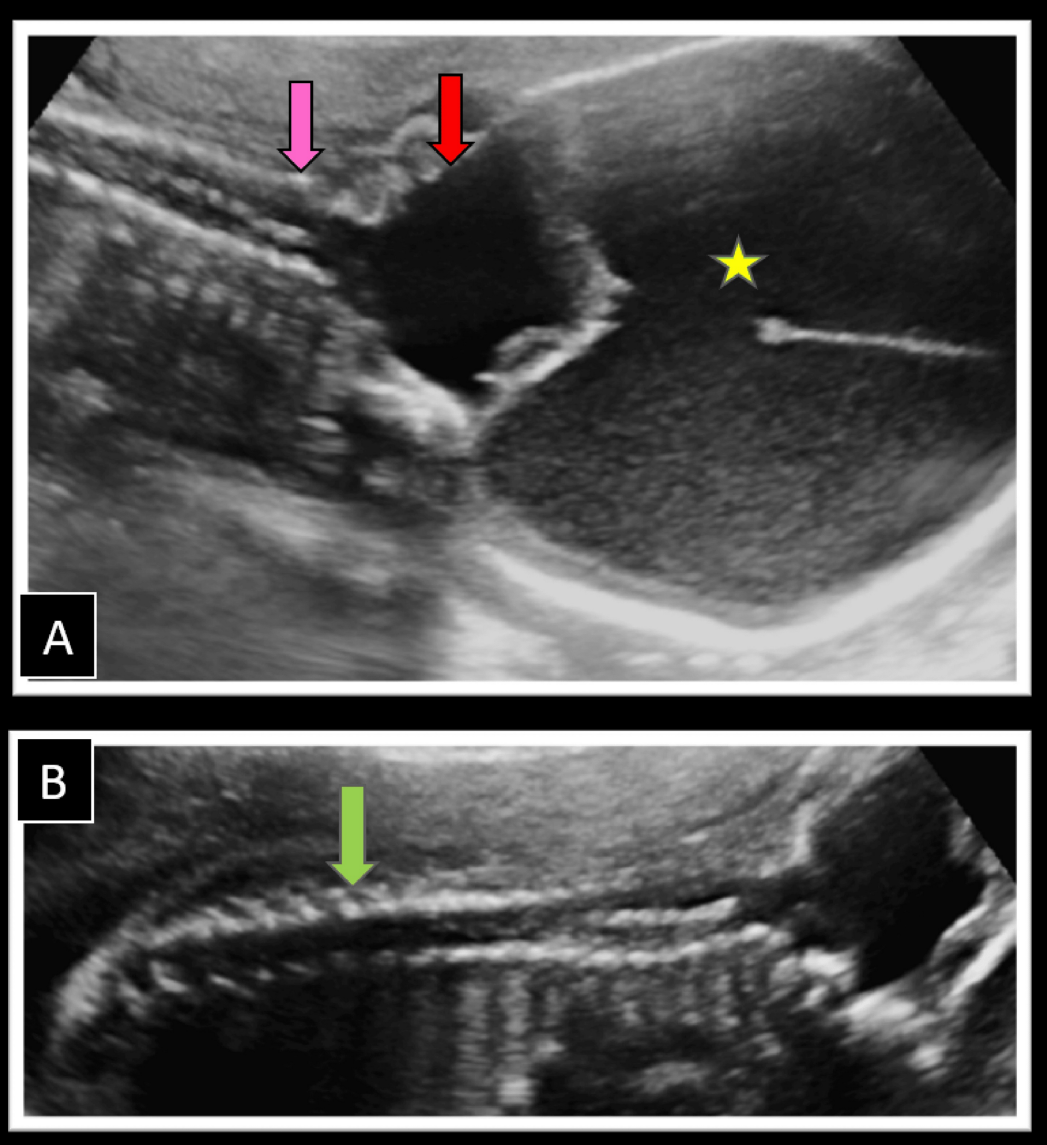
Ultrasound examination of the fetal spine (A) Coronal image of the fetal cervical spine showing normal posterior elements (pink arrow) with no evidence of a bony defect, encephalocele, or herniation of brain tissue into the spinal canal. Also note the enlarged posterior fossa cerebrospinal fluid cyst (red arrow) and gross hydrocephalus (star). (B) The normal thoracic and lumbar vertebral column is seen with no evidence of spinal dysraphism or meningocele (green arrow).

On the evaluation of the central nervous system, an enlarged posterior fossa with a cerebrospinal fluid (CSF) cyst, communicating with the fourth ventricle with severely hypoplastic cerebellar hemispheres and absent cerebellar vermis was noted (Figure 1A), favoring the diagnosis of DWM. This was associated with communicating hydrocephalus with enlarged lateral ventricles (right 15 mm, left 16 mm in width at the level of atrium), paper-thin overlying parenchyma, and dilated third ventricle (Figure 1B). Midline falx was visualized with normal bilateral thalami showing no evidence of fusion, ruling out the possibility of holoprosencephaly. Cavum septum pellucidum was normally formed with no evidence of corpus callosum agenesis. There was no evidence of a spinal column deformity, meningocele, or herniation of posterior fossa structures ruling out the possibility of Arnold Chiari malformation (Figures 2A, 2B). No occipital encephalocele was noted. Orbits, nose, lips, and palate were normally formed.

**Figure 3:**
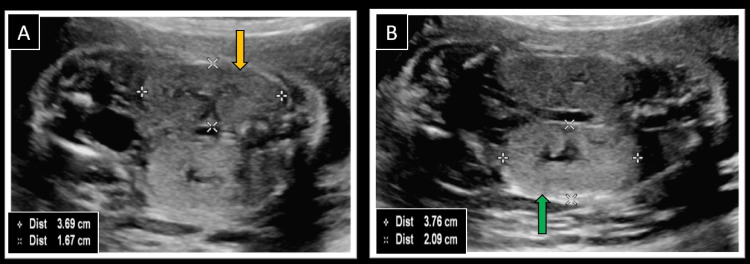
Ultrasound images showing measurements of fetal kidneys Coronal ultrasound images showing length and width measurements of (A) Right fetal kidney (yellow arrow) = 3.69 x 1.67 cm and (B) Left fetal kidney (green arrow) = 3.76 x 2.09 cm, both of which were enlarged for gestational age (Z-score greater than two standard deviations) [7]. Note the normal shape and contour of both kidneys, with no evidence of any mass lesion or cyst.

**Figure 4:**
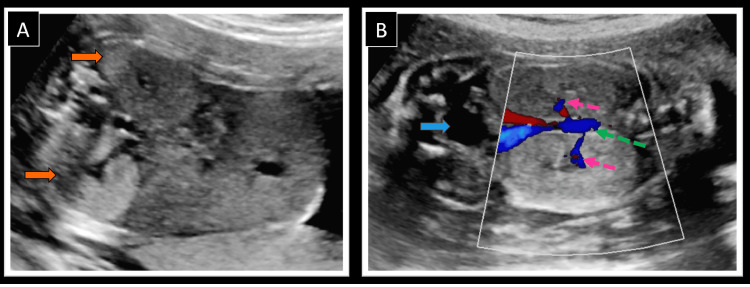
Fetal abdominal ultrasound showing bilateral enlarged dysplastic kidneys (A) Transverse ultrasound image through the fetal abdomen showing symmetrically enlarged and echogenic bilateral kidneys (orange arrows). (B) Coronal color Doppler ultrasound image showing abdominal aorta (green arrow) giving off two normal caliber renal arteries (pink arrows) entering renal hilum. A partially distended urinary bladder is also seen in the image (blue arrow).

Examination of the abdomen revealed bilateral symmetrically hyperechoic and enlarged kidneys (Z score above two standard deviations above the mean value for GA) (Figure 3A, 3B) [7]. No cysts were seen in the kidneys; both the renal arteries were normal (Figures 4A, 4B). A partially distended fetal urinary bladder was visualized. The amniotic fluid volume was low (amniotic fluid index - 4 cm), suggesting reduced urine production by the kidneys and indicating the possibility of dysplastic kidneys. All four limbs of the fetus were normal with no evidence of polydactyly. A normal three-vessel umbilical cord was seen. No other abnormality was detected on thorough fetal examination.

**Figure 5:**
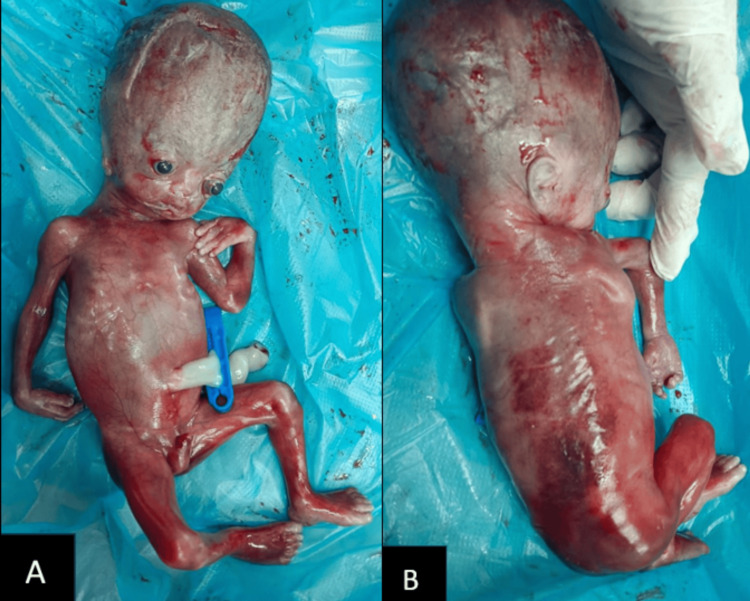
Male fetus delivered at 22 weeks of gestational age with Goldston syndrome (A) A male fetus with an enlarged head due to hydrocephalus was delivered at 22 weeks of gestational age. There was no evidence of any facial anomalies, polydactyly, or limb deformities. (B) The spinal column was normal externally with no meningocele or encephalocele. A normal neck and right ear are also seen in the image.

Based on the ultrasound findings, a diagnosis of DWM with bilateral enlarged echogenic kidneys and oligohydramnios was made with the possibility of GS. The patient was counseled about the poor prognosis of the fetus, and she decided to undergo elective termination of pregnancy. A male fetus was delivered with an enlarged head suggestive of hydrocephalus (Figures 5A, 5B). Genetic counseling and pre-conceptional folic acid treatment were advised, and the patient was discharged.

## Discussion

GS is a cerebro-renal syndrome characterized by the concomitant occurrence of cystic dysplastic kidneys and DWM [3]. Both these conditions have a familial association with an autosomal recessive inheritance and their association is extremely rare [3]. DWM is detected on antenatal ultrasound by the presence of a triad of enlarged posterior fossa, vermian agenesis/dysgenesis, and large posterior fossa cyst communicating with the fourth ventricle [2,3,8]. The presence of oligohydramnios with bilateral, symmetrically enlarged, and echogenic fetal kidneys raises the possibility of dysplastic and polycystic kidneys [3,8]. This syndrome was first reported by Goldston in 1963 [8] in three siblings postnatally while the first antenatal case was reported by Gloeb et al. [9] in a male fetus with DWM and bilateral echogenic kidneys and microscopic features of hepatic fibrosis at the seventeenth week of gestation in 1989. In 1993 Moerman et al. reported two siblings born with a combination of hepatic ductal plate malformation along with central nervous system malformation and renal dysplasia [10]. One sibling was diagnosed with GS while the other had an occipital encephalocele and was diagnosed with MGS [10]. This raised the question of whether GS is a separate entity or just a less severe variant of MGS [1,10].

MGS is a rare and lethal autosomal recessive disorder, which is the prototype of congenital cerebro-renal malformations [1,11,12]. It is characterized by cystic renal dysplasia, occipital encephalocele, and post-axial polydactyly affecting all four limbs [5,13]. However, all three cardinal features are found in only about half of the cases [5] and at least two of these three anomalies are required to make the diagnosis of MGS [14]. A variety of other malformations have been reported in MGS including DWM, cystic hygroma, cardiac malformation, single umbilical artery, several genital abnormalities, oligohydramnios, microcephaly, intrauterine growth retardation, and cleft palate [1,5]. Hepatic fibrosis and bile duct proliferation can be present in up to one-fifth of the cases [1]. The condition has a very poor prognosis with most pregnancies ending in stillbirth, abortion, or death soon after birth [1]. Our case had no evidence of occipital encephalocele or occipital bone defect and all four limbs were normal with normal finger count. Walpole et al. reported a family in which three nonviable brothers had DWM (variant) with associated enlarged cystic renal dysplastic kidneys and hepatic fibrosis [15]. In the absence of polydactyly and encephalocele, Walpole et al. suggested the possibility of a separate and distinct syndrome [15,16]. Miranda syndrome is another rare familial cerebro-renal syndrome associated with DWM, congenital hepatic fibrosis, and generalized cystic dysplastic renal lesions [3,12].

Very few antenatally diagnosed cases of GS have been reported in the literature including those reported by Gulcan et al. [17] in 2001 at 28 weeks of GA, by Avcu et al. [4] in 2011 at 22 weeks of gestation with hydrocephalus [3], and by Hussain et al. [2] in 2011 at 27 weeks of gestation with oligohydramnios. In 2012, Al‑Shahrani [11] and in 2015, Rathod et al. [1] described antenatal cases of GS associated with massive fetal ascites. In 2020, Kayastha et al. [3] reported a case at 19 weeks of GA. In 2016, Nagabhushan BM et al. [18], and in 2019, Agrwal et al. [5] reported GS in a neonate, associated with congenital hepatic fibrosis in the latter case. Menon et al.[16] reported the first case of this syndrome in an adult patient in 2005. A 32‑year‑old woman with bilateral subdural hematomas and bilateral large cystic kidneys was found to have DWM with hypo‑plastic vermis [1,16].

Our case was detected antenatally at 22 weeks of GA with no history of renal anomalies in parents or congenital anomalies in prior pregnancies. The fetus was found to have DWM with hydrocephalus and mild oligohydramnios with bilateral echogenic enlarged kidneys suggestive of dysplastic kidneys with a possibility of autosomal recessive polycystic kidney disease, likely to be suffering from GS. The history of consanguineous marriage favored autosomal recessive inheritance of the disease. Less than 20 cases of antenatally detected Goldston syndrome have been reported in global literature, and our case is one of these rare few. Our case also highlights the importance of early detection of this syndrome to allow for proper counseling of the parents giving them an option of termination of pregnancy due to the poor prognosis of the syndrome.

## Conclusions

GS is a rare congenital syndrome with a poor prognosis, making timely antenatal diagnosis extremely important. The key to identifying this syndrome is detecting the association of DWM (characterized by agenesis/dysgenesis of the vermis and an enlarged posterior fossa cyst communicating with the fourth ventricle) with bilateral enlarged, echogenic, and dysplastic kidneys. When these features are detected in a fetus, all four limbs should be evaluated to look for the presence of post-axial polydactyly and the head and neck should be evaluated to look for occipital encephalocele; the presence of either of these features changes the diagnosis to MGS. A thorough analysis of the fetal cardiovascular and genitourinary systems, along with evaluation for intrauterine growth retardation is also recommended, as these syndromes may be associated with a variety of other anomalies.

Proper parental counseling is needed about the management of the pregnancy, the option of elective abortion, risks of pregnancy complications like stillbirth, complications and risks to the child post-delivery, and postnatal care if parents choose to continue with the pregnancy. Also, parents need to undergo genetic counseling to manage the possibility of recurrence of this syndrome in future pregnancies.
